# Effect of end-stage renal disease on long-term survival after a first-ever mechanical ventilation: a population-based study

**DOI:** 10.1186/s13054-015-1071-x

**Published:** 2015-10-01

**Authors:** Chin-Ming Chen, Chih-Cheng Lai, Kuo-Chen Cheng, Shih-Feng Weng, Wei-Lun Liu, Hsiu-Nien Shen

**Affiliations:** Department of Recreation and Health-Care Management, Chia Nan University of Pharmacy and Science, No.60, Sec. 1, Erren Road., Rende District, Tainan, 71710 Taiwan; Department of Hospital and Health Care Administration, Chia Nan University of Pharmacy and Science, Tainan, Taiwan; Southern Taiwan University of Science and Technology, No. 1, Nan-Tai Street, Yungkang District, Tainan, 710 Taiwan; Department of Intensive Care Medicine, Chi Mei Medical Center, 901 Chung Hwa Road, Yungkang District, Tainan, 710 Taiwan; Department of Internal Medicine, Chi Mei Medical Center, 901 Chung Hwa Road, Yungkang District, Tainan, 710 Taiwan; Department of Healthcare Administration and Medical Informatics, Kaohsiung Medical University, Kaohsiung, Taiwan; Department of Intensive Care Medicine, Chi Mei Medical Center, Liouying District, 201, Taikang, Taikang Village, Liouying District, Tainan, 736 Taiwan; Department of Safety, Health and Environment, Chung Hwa University of Medical Technology, 89, Wenhua 1st Street, Rende District, Tainan, 717 Taiwan; Department of Public Health, College of Medicine, National Cheng Kung University, 1, University Road, Tainan, 701 Taiwan

## Abstract

**Introduction:**

Patients with end-stage renal disease (ESRD^Pos^) usually have multiple comorbidities and are predisposed to acute organ failure and in-hospital mortality. We assessed the effect of ESRD on the poorly understood long-term mortality risk after a first-ever mechanical ventilation (1-MV) for acute respiratory failure.

**Methods:**

The data source was Taiwan’s National Health Insurance (NHI) Research Database. All patients given a 1-MV between 1999 and 2008 from one million randomly selected NHI beneficiaries were identified (*n* = 38,659). Patients with or without ESRD (ESRD^Neg^) after a 1-MV between 1999 and 2008 were retrospectively compared and followed from the index admission date to death or the end of 2011. ESRD^Pos^ patients (*n* = 1185; mean age: 65.9 years; men: 51.5 %) were individually matched to ESRD^Neg^ patients (ratio: 1:8) using a propensity score method. The primary outcome was death after a 1-MV. The effect of ESRD on the risk of death after MV was assessed. A Cox proportional hazard regression model was used to assess how ESRD affected the mortality risk after a 1-MV.

**Results:**

The baseline characteristics of the two cohorts were balanced, but the incidence of mortality was higher in ESRD^Pos^ patients than in ESRD^Neg^ patients (342.30 versus 179.67 per 1000 person-years; *P* <0.001; covariate-adjusted hazard ratio: 1.43; 95 % confidence interval: 1.31–1.51). For patients who survived until discharge, ESRD was not associated with long-term (>4 years) mortality.

**Conclusions:**

ESRD increased the mortality risk after a 1-MV, but long-term survival seemed similar.

**Electronic supplementary material:**

The online version of this article (doi:10.1186/s13054-015-1071-x) contains supplementary material, which is available to authorized users.

## Introduction

End-stage renal disease (ESRD) is becoming more common worldwide, especially in Taiwan. Since 2000, the incidence and prevalence of ESRD have increased [1]. According to the United States Renal Data System, Taiwan had the highest incidence (418 per million population) and prevalence (2226 cases per million population) of patients on chronic dialysis between 2000 and 2007 [2]. From 2006 to 2010, the number of patients on chronic dialysis increased from 52,081 to 65,883 (+26.5 %) [3, 4]. Because of their frequent multiple comorbidities, patients with ESRD (ESRD^Pos^) are prone to developing acute critical illnesses and have a higher mortality than do those without ESRD (ESRD^Neg^) [5–11]. However, whether ESRD per se increases the risk of short-term and long-term mortality after critical illnesses remains controversial [12, 13]. For example, several small cohort studies report that critically ill patients with ESRD have a higher risk of short-term mortality than do those without ESRD [8, 14, 15], and preexisting chronic kidney disease (CKD) indeed has a marked effect on the development of acute renal failure, 30-day, and 1-year mortality in critically ill patients who require mechanical ventilation (MV) [16]. Others, however, have found different results [7, 13]. One large cohort study showed that critically ill ESRD^Pos^ patients had a similar 1-year mortality rate to those without kidney dysfunction after age, illness severity, and admission type had been adjusted for [13]. Presumably, the observed difference in mortality in other studies is largely because of differences in the burden of comorbidities and the severity of acute illnesses rather than because of ESRD [7, 13]. Given these conflicting results, additional research on longer-term outcomes of these patients is needed.

MV is a life-support measure for patients with acute respiratory failure (ARF), who have a high rate of in-hospital mortality (up to 35 %) [17]. When ESRD^Pos^ patients undergo MV, there is a high rate of weaning failure and mortality [18, 19]. Moreover, when MV is prolonged, the outcomes for these patients become even worse, with 1-year mortality as high as 60 % [20]. However, whether ESRD predicts a worse long-term outcome in patients who undergo MV is not known.

Studies on critically ill ESRD^Pos^ patients focus primarily on those admitted to the intensive care unit (ICU) [6, 11]. However, not all patients with critical illnesses are admitted to the ICU [21, 22]. Therefore, these studies usually have a selection bias. Moreover, ordering an invasive MV is usually a major critical decision for life-support measures in ESRD^Pos^ patients, whether or not they are admitted to the ICU. Information on the effects of ESRD on patients who undergo MV, instead of those admitted to the ICU, would be more relevant for patients. Therefore, we investigated the long-term outcomes of ESRD^Pos^ patients who underwent their first-ever MV (1-MV).

## Methods

### Data source

The data used in this study are from the National Health Insurance Research Database (NHIRD) established by Taiwan’s National Health Research Institute (NHRI) to improve medical research. Taiwan’s NHI program, instituted in March 1995, provides coverage for more than 99 % of the country’s legal residents; therefore, the NHIRD offers detailed healthcare services information on clinical visits for each insured beneficiary, using *International Classification of Diseases, Ninth Revision, Clinical Modification* (ICD-9-CM) diagnostic and procedure codes [23]. In the present study, data used came from the Longitudinal Health Insurance Database 2000 (LHID2000) [24], a sub-dataset of the NHI program, which contains all claims data, from 1996 to 2011, of one million NHI beneficiaries (about 4.34 % of the total population) who were randomly selected from the year 2000 Registry of Beneficiaries of the NHIRD. There are no significant differences in gender distribution between beneficiaries in the LHID2000 and the NHIRD. The study was done according to the Declaration of Helsinki and was approved by the Institutional Review Board (IRB) at Chi Mei Medical Center (10308-E01). The IRB waived the need for informed consent from the enrolled participants because the data used in this study consists of nationwide, de-identified, secondary data released to the public for research purposes. This waiver does not adversely affect the rights and welfare of the enrollees.

### Patient selection and definition

We enrolled all inpatients with a 1-MV for ARF during their first hospitalization between 1999 and 2008 (*n* = 38,659). ESRD^Pos^ patients (ICD-9-CM code 585) were detected using the NHI’s catastrophic illness certification records, which included those who had undergone regular dialysis for at least 3 months. Those who were diagnosed with ESRD after a 1-MV were excluded (*n* = 1,013). The enrolled ESRD^Pos^ patients (*n* = 1,331) were then, using propensity score matching and the greedy matching algorithm (without replacement), individually matched to ESRD^Neg^ controls in a 1:8 ratio. The propensity score, i.e., the probability of being ESRD^Pos^, was estimated using a logistic regression model conditional on the covariates age, sex, length of ICU stay, length of hospital stay, duration of MV, department to which admitted, number of organ failures (other than respiratory and renal systems) [25], and individual comorbidities: diabetes mellitus (DM), hypertension (HTN), coronary artery disease (CAD), cirrhosis, chronic obstructive pulmonary disease (COPD), cancer, stroke, and congestive heart failure (CHF) (Additional file 1). Propensity score matching was used to reduce selection bias because it can bundle many confounding covariates that might be present in an observational study with this number of variables. The characteristics of the two groups were balanced after the propensity score matching (Table 1).

**Table 1:** Baseline characteristics of the study participants before and after propensity score matching

	Before propensity score matching			After propensity score matching		
Variables	ESRD^Pos^	ESRD^Neg^	*P*	ESRD^Pos^	ESRD^Neg^	*P*
Total	1331 (3.54)	36,315 (96.46)		1185 (11.11)	9480 (88.89)	
Age, years (mean ± SD)	65.02 ± 12.97	62.84 ± 20.65	<0.0001	65.90 ± 13.47	65.48 ± 14.99	0.3005
Age group, years			<0.0001			0.8569
<50	159 (11.95)	8454 (23.28)		152 (12.83)	1264 (13.33)	
50–64	422 (31.71)	7079 (19.49)		339 (28.61)	2731 (28.81)	
≧65	750 (56.35)	20,782 (57.23)		694 (58.57)	5485 (57.86)	
Sex			<0.0001			0.2491
Female	689 (51.77)	13,407 (36.92)		575 (48.52)	4432 (46.75)	
Male	642 (48.23)	22,908 (63.08)		610 (51.48)	5048 (53.25)	
Comorbidity						
Diabetes	703 (52.82)	9779 (26.93)	<0.0001	568 (47.93)	4379 (46.19)	0.2573
Hypertension	903 (67.84)	15,755 (43.38)	<0.0001	757 (63.88)	5954 (62.81)	0.4697
CAD	458 (34.41)	8173 (22.51)	<0.0001	377 (31.81)	2948 (31.10)	0.6153
Liver cirrhosis	137 (10.29)	2954 (8.13)	0.0048	120 (10.13)	1025 (10.81)	0.4722
COPD	175 (13.15)	8715 (24.00)	<0.0001	175 (14.77)	1394 (14.70)	0.9538
Cancer	184 (13.82)	7912 (21.79)	<0.0001	183 (15.44)	1475 (15.56)	0.9172
Stroke	368 (27.65)	9952 (27.40)	0.8448	343 (28.95)	2711 (28.60)	0.8027
CHF	279 (20.96)	5024 (13.83)	<0.0001	215 (18.14)	1699 (17.92)	0.8514
Department to which admitted			<0.0001			0.6634
Surgery	325 (24.42)	14,358 (39.54)		324 (27.34)	2649 (27.94)	
Medical	1006 (75.58)	21,957 (60.46)		861 (72.66)	6831 (72.06)	
Number of organ failures (other than lungs and kidneys)			<0.0001			0.8884
0	960 (72.13)	28,900 (79.58)		867 (73.16)	6979 (73.62)	
1	346 (26.00)	6779 (18.67)		294 (24.81)	2298 (24.24)	
≧2	25 (1.88)	636 (1.75)		24 (2.03)	203 (2.14)	
Ventilator duration (days) (continuous)	11.69 ± 25.34	18.15 ± 84.99	<0.0001	11.57 ± 25.23	11.93 ± 29.57	0.6494
ICU days, mean ± SD	10.25 ± 16.48	9.75 ± 17.18	0.2839	9.93 ± 15.32	10.07 ± 17.18	0.7883
Hospital days, mean ± SD	24.06 ± 28.14	25.28 ± 54.04	0.1391	23.92 ± 28.21	22.89 ± 32.11	0.2444

Data are number (percentages) unless otherwise specified

*ESRD* end stage renal disease, *ESRD* ^*Pos*^ patients with ESRD, *ESRD* ^*Neg*^ patients without ESRD, *CAD* coronary artery disease, *COPD* chronic obstructive airway disease, *CHF* congestive heart disease, *ICU* intensive care unit

### Endpoint

The primary endpoint (outcome) of the study was death after MV. Patients were followed from the index admission date to death or to the end of 2011. The secondary endpoint was to identify the risk factors for all-cause mortality after a 1-MV. We hypothesized that mortality is higher in ESRD^Pos^ patients than in ESRD^Neg^ patients who require MV. The demographic and clinical characteristics of age; sex; length of hospital stay, length of ICU stay, and duration of MV; department to which admitted; number of organ failures; and comorbidities were used to estimate the mortality risk.

### Statistical analysis

Differences in baseline characteristics between groups were evaluated using Pearson’s χ^2^ test for categorical variables and Student’s *t* test for continuous variables. The incidence rate (IR) of death was calculated as cases per person-year. The overall and subgroup-specific relative mortality risks between the two groups were estimated using the incidence rate ratio (IRR) with a 95 % confidence interval (CI) using the Poisson assumption. The actuarial survival rate of the two groups was determined using the Kaplan-Meier method, and a log-rank test was used to compare the difference between the two survival curves. The effect of ESRD on the mortality risk after MV was assessed using a Cox proportional hazards regression model. Covariates included in the Cox model were those used in the propensity score matching (mentioned in the “Patient selection and definition” subsection above). The proportional hazard assumption was verified using plots of natural log transformed (ln) (survival function) versus ln (time). The data are mean ± standard deviation or number (percentages). Significance was set at *P* <0.05. SAS 9.3.1 for Windows (SAS Institute, Cary, NC, USA) was used for all analyses.

## Results

The initial survey included 37,646 patients. After matching, 10,665 patients with a 1-MV (ESRD^Pos^: 1185 and ESRD^Neg^: 9480) were selected. Before propensity score matching, the ESRD^Pos^ group contained more patients who were older, female, and had comorbid DM, HTN, CAD, cirrhosis, and CHF, and contained fewer patients who had COPD and cancer, a higher prevalence of one or more organ failures, and fewer days on MV than did ESRD^Neg^ patients (Table 1). In addition, about 12.17 % (162/1331) of the ESRD^Pos^ patients and 9.76 % (3545/36,315) of the ESRD^Neg^ patients had undergone MV outside the ICU (data not shown). After propensity score matching, with the correction of all the above variables, 1185 (90.39 %) of the 1331 ESRD^Pos^ patients were matched to 9480 ESRD^Neg^ controls (Table 1). The mortality rate of ESRD^Pos^ patients was nearly twice as high as that of ESRD^Neg^ patients (IRR = 1.92) (Table 2). The risk difference in mortality between the two groups was significant across all subgroups with the exception of those with liver cirrhosis, cancer, and multiple organ failure. The highest risk difference was for patients admitted to the Surgery Department (IRR = 2.95), and the lowest was for patients with CHF (IRR = 1.35). In follow-ups within 4 years, ESRD^Pos^ patients had a higher mortality rate (IRR, 0–6 months: 1.59; 6–12 months: 1.84; 1–2 years: 1.65 and 2–4 years: 1.69). After 4 years of follow-up, however, there was no significant difference in mortality rates between ESRD^Pos^ and ESRD^Neg^ patients. The 30-day, 6-month, and 1-, 2-, 5-, and 10-year survival rate differences in the ESRD^Pos^ and ESRD^Neg^ groups from the beginning are listed in Additional file 2.

**Table 2:** The overall and subgroup-specific incidence rates (IR) and incidence rate ratios (IRR) of death between ESRD^Pos^ patients and matched ESRD^Neg^ controls

Variables	ESRD^Pos^ patients			ESRD^Neg^ controls			IRR (95 % CI)	*P*
	Total (*n*)	Death (n)	IR (per 1000 person-years)	Total (*n*)	Death (n)	IR (per 1000 person-years)		
All	1185	898	342.30	9480	5806	179.67	1.92 (1.79–2.07)	<0.0001
Age group, years								
<50	152	90	175.34	1264	509	80.90	2.17 (1.73–2.71)	<0.0001
50–64	339	241	261.52	2731	1481	142.16	1.84 (1.61–2.11)	<0.0001
≧65	694	567	477.04	5485	3816	244.53	1.95 (1.79–2.13)	<0.0001
Sex								
Male	610	474	388.34	5048	3102	179.79	2.16 (1.96–2.38)	<0.0001
Female	575	424	302.25	4432	2704	179.64	1.68 (1.52–1.86)	<0.0001
Comorbidity								
Diabetes	568	447	411.98	4379	2946	224.98	1.83 (1.66–2.02)	<0.0001
Hypertension	757	570	336.70	5954	3764	193.81	1.74 (1.59–1.90)	<0.0001
CAD	377	293	386.34	2948	1830	181.80	2.13 (1.88–2.40)	<0.0001
Liver cirrhosis	120	101	481.91	1025	839	449.78	1.07 (0.87–1.32)	0.5123
COPD	175	153	602.27	1394	960	219.92	2.74 (2.31–3.25)	<0.0001
Cancer	183	138	339.96	1475	1068	294.69	1.15 (0.97–1.38)	0.1142
Stroke	343	278	463.19	2711	1852	236.90	1.96 (1.72–2.22)	<0.0001
CHF	215	161	338.72	1699	1179	251.31	1.35 (1.14–1.59)	0.0004
Department to which admitted								
Surgery	324	225	257.56	2649	1133	87.26	2.95 (2.56–3.41)	<0.0001
Medical	861	673	384.60	6831	4673	241.73	1.59(1.47–1.72)	<0.0001
Number of organ failures (other than lungs and kidneys)								
0	867	633	290.32	6979	3812	136.93	2.12 (1.95–2.31)	<0.0001
1	294	245	589.45	2298	1818	430.62	1.37 (1.20–1.56)	<0.0001
≧2	24	20	728.53	203	176	691.46	1.05 (0.66–1.67)	0.8249
Follow-up								
0–6 month	1185	717	2497.50	9480	4445	1571.80	1.59 (1.47–1.72)	<0.0001
6–12 months	468	44	197.37	5035	263	107.515	1.84 (1.33–2.53)	0.0002
1–2 years	424	46	115.34	4772	321	69.83	1.65 (1.21–2.25)	0.0015
2–4 years	378	52	76.36	4451	374	45.28	1.69 (1.26–2.25)	0.0004
4–6 years	292	22	45.21	3616	186	30.25	1.49 (0.96–2.32)	0.0747
> = 6 years	196	17	31.08	2537	217	27.01	1.15 (0.70–1.89)	0.5570

*ESRD* end stage renal disease, *ESRD* ^*Pos*^ with ESRD, *ESRD* ^*Neg*^ without ESRD, *IRR* incidence rate ratio, *CI* confidence interval, *IR* incidence rate, *CAD* coronary artery disease, *COPD* chronic obstructive airway disease, *CHF* congestive heart disease

**Fig. 1:**
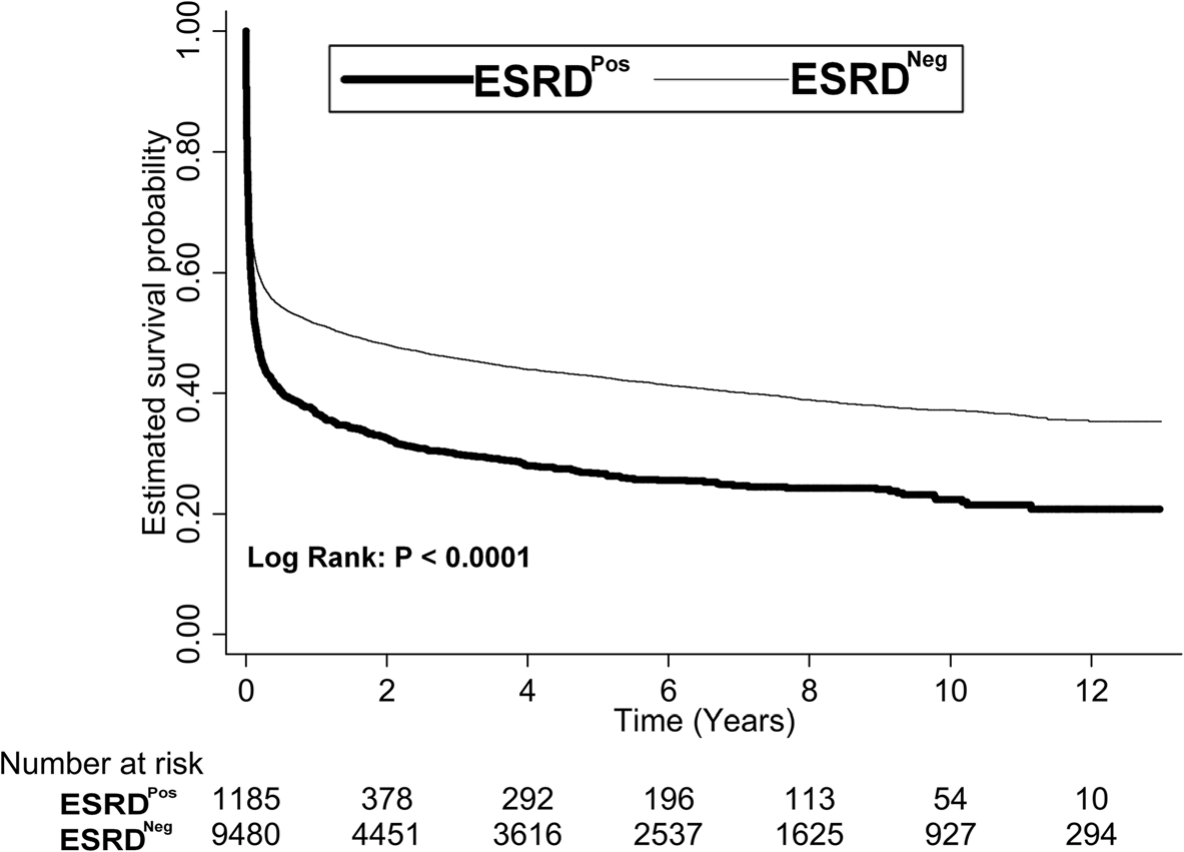
Kaplan-Meier survival curves of ESRD^Pos^ patients and ESRD^Neg^ controls. *ESRD* end stage renal disease

**Fig. 2:**
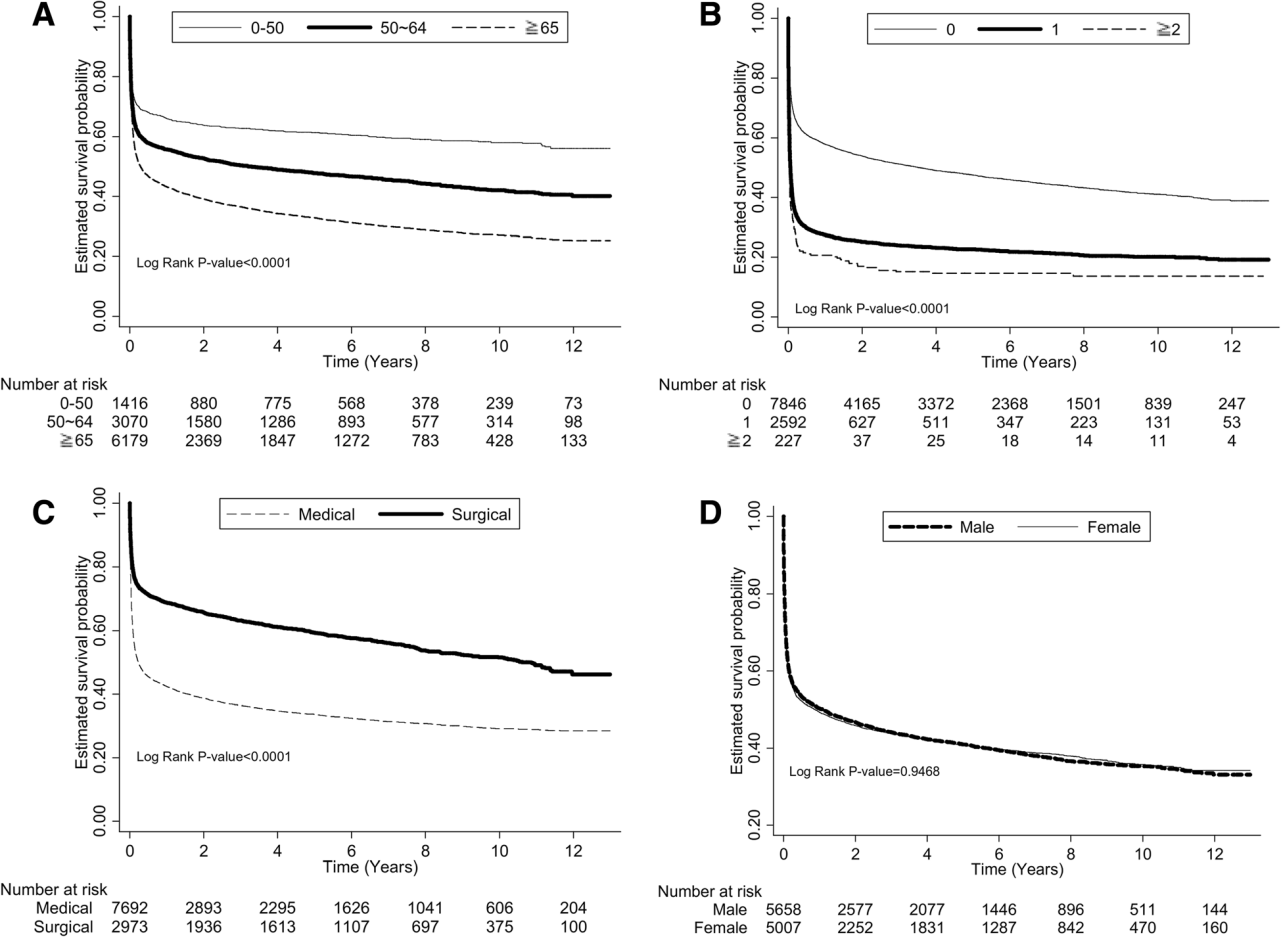
Kaplan-Meier survival curves of different groups: (**a**) age; (**b**) number of organ failures; (**c**) departments to which patients were admitted; (**d**) sex

ESRD^Pos^ patients after a 1-MV showed a precipitous decline in mortality early on, and a parallel course thereafter, which suggests that although the starting point is lower, the trajectory has not changed (Fig. 1). After 4 years, the survival curves seem to be almost parallel, which might indicate that ESRD increases short-term but not long-term mortality. Patients who were older, had more organ failures, and had been admitted to the Surgery Department had a significantly higher mortality (Fig. 2a–c), but there was no significant difference in the survival rate between males and females (Fig. 2d).

ESRD^Pos^ patients had a lower survival rate than did ESRD^Neg^ patients (hazard ratio [HR] 1.43; 95 % CI: 1.33–1.54). In addition to ESRD, some important factors predicting mortality for patients after a 1-MV included older age (50–64 years old: HR = 1.50; ≥65 years old: HR = 2.03: compared with 0–50 years old), being female (HR = 0.94), admitted to a surgery department (0.64), more organ failures (1: HR = 1.85; ≥2: HR = 2.46: compared with 0), DM (HR = 1.18), liver cirrhosis (HR = 1.61), cancer (1.49), stroke (1.26), and CHF (1.17) (Table 3). The HR of death in ESRD^Pos^ patients treated with MV is shown in Additional file 3.

**Table 3:** Crude and adjusted hazard ratios (HR) of death in all ventilated patients (derived using Cox proportional hazard regression models)

Cohort	Crude HR (95 % CI)	Adjusted HR (95 % CI)
ESRD		
Yes	1.47 (1.36–1.57)	1.43 (1.33–1.54)
No	1.00	1.00
Age (years)		
0–50	1.00	1.00
50–64	1.51 (1.37–1.66)	1.50 (1.36–1.65)
≧65	2.18 (1.99–2.38)	2.03 (1.85–2.23)
Sex		
Female	1.00 (0.95–1.05)	0.94 (0.89–0.99)
Male	1.00	1.00
Comorbidity		
Diabetes	1.30 (1.24–1.36)	1.18 (1.13–1.25)
Hypertension	1.10 (1.05–1.16)	0.99 (0.94–1.05)
Coronary artery disease	1.00 (0.95–1.06)	0.93 (0.88–1.01)
Liver cirrhosis	1.90 (1.77–2.04)	1.61 (1.50–1.74)
COPD	1.20 (1.12–1.28)	1.01 (0.95–1.08)
Cancer	1.33 (1.24–1.41)	1.49 (1.39–1.59)
Stroke	1.28 (1.21–1.34)	1.26 (1.19–1.33)
CHF	1.24 (1.17–1.32)	1.17 (1.10–1.25)
Department to which admitted		
Surgery	0.50 (0.47–0.53)	0.64 (0.60–0.68)
Medical	1.00	1.00
Number of organ failures (other than lungs and kidneys)		
0	1.00	1.00
1	2.14 (2.03–2.26)	1.85 (1.75–1.95)
≥2	2.79 (2.41–3.23)	2.46 (2.12–2.86)
Ventilator duration (days) (continuous)	1.00 (1.00–1.00)	1.00 (1.00–1.00)
ICU stay (days) (continuous)	1.00 (1.00–1.00)	1.00 (1.00–1.01)
Hospital stay (days) (continuous)	0.99 (0.99–1.00)	1.00 (0.99–1.00)

*HR* hazard ratio, *CI* confidence interval, *ESRD* end-stage renal disease, *COPD* chronic obstructive airway disease, *CHF* congestive heart disease, *ICU* intensive care unit

## Discussion

We found that after age, severity of illness, comorbidities, and department to which admitted had been adjusted for, ESRD after a 1-MV was associated with a higher risk for mortality (HR = 1.43), regardless of whether the patient had been admitted to the ICU. We also found that about 9.85 % (3707/37,646) of ESRD^Pos^ 1-MV patients had never been admitted to the ICU (data not shown), which is consistent with other reports [21, 22]. The present study appears to be the first to describe long-term outcomes of ESRD^Pos^ patients with a 1-MV and to include patients not admitted to the ICU. Our findings are compatible with most other studies on ESRD^Pos^ patients admitted to the ICU. One study of 276,731 adults admitted to the ICU reported that after the patients had been discharged from the ICU, in-hospital mortality rates were much higher in ESRD^Pos^ patients than in ESRD^Neg^ patients (45.3 % versus 31.2 %; *P* <0.001) [9].

Go et al. also showed that ESRD^Pos^ patients have a relative risk for all-cause mortality 5.9 times greater than do patients with healthy renal function [26]. Another study reported that ESRD^Pos^ patients had higher rates of ICU and in-hospital mortality than did matched pairs of patients (23.1 % versus 15.1 %, and 31.2 % versus 19.1 %, *P* <0.05) [10]. Other studies have reported that critically ill patients on chronic dialysis are estimated to have the following mortality rates: in-hospital: 14 % to 56 %, 30-day: 32 % to 41 %, and 90-day: 42 % to 44.6 % [6], and that longer-term mortality rates might be as high as 38 % (6 months) and 48 % (12 months) [27]. There are few studies on the long-term outcomes of ESRD^Pos^ patients after a 1-MV, except for one on 47 patients, which reported an overall cumulative proportional in-hospital survival rate of only 17 %, a 1-year rate of 40 %, and a 3-year rate of 25 % [18]. Liao et al. also said that ESRD^Pos^ patients had a significantly higher mortality rate than did ESRD^Neg^ patients (76.7 % versus 28.8 %) 1 year after traumatic brain injury [28]. Those studies were exclusively on patients admitted to the ICU, however.

In contrast, others have found different results. A large population-based cohort study of nonspecific critically ill patients (5693 admissions) showed that any kidney dysfunction is associated with an increased risk for long-term death, with the exception of ESRD^Pos^ patients, who had outcomes similar to those of patients with no kidney dysfunction [13]. Strijack et al. [7] said that the unadjusted in-hospital mortality rate was higher for ESRD^Pos^ patients (16 % versus 11 %), but that this difference did not persist after an adjustment for baseline illness severity, and that the higher mortality rate was due to comorbidity but not to ESRD itself. Moreover, Chapman et al. [29] reported that ESRD^Pos^ patients who were alive after they had been discharged from the ICU had a 2-year survival rate of 56 %, but that the long-term mortality rate between ESRD^Pos^ patients and matched ESRD^Neg^ controls was similar after excluding patients who had died within a month of being discharged from the ICU [29]. We thought that because of different inclusion criteria, critically ill ESRD^Pos^ patients might have different long-term outcomes because they have different comorbidities. Despite possible bias, including possible MV patients not admitted to the ICU, and matching using propensity scores, our study showed that ESRD^Pos^ patients with a 1-MV had a higher IRR of death per 1000 person-years than did almost all the groups (stratified by age, sex, department admitted by, number of organ failures, and comorbidities) and a higher covariate-adjusted HR (1.43) than did ESRD^Neg^ controls. The long-term outcomes and the long-term mortality rates of those who survived for more than 4 years after they had been discharged from the hospital were similar between ESRD^Pos^ and ESRD^Neg^ patients.

After a Cox proportional hazards analysis, we showed that the mortality predictors of our patients treated with MV included ESRD, older age, and being male, findings consistent with the literature. Other researchers have reported that ESRD predicted in-hospital or 30-day mortality in patients with ARF and MV admitted to the ICU in spite of aggressive treatment [19, 30], and that for patients with prolonged MV, ESRD also predicted 1-year mortality [20]. Moreover, many studies have reported the effect of age on the mortality of patients treated with MV [13, 20, 30–34]. For example, a retrospective study [34] of 61,113 patients treated with MV showed that factors independently associated with an increased mortality rate included being >80 years old, and two recent studies also reported that older age was a consistent hazard [30, 32]. Esteban et al. [31], too, said that older age (40–70 years: HR = 1.60 and >70 years: HR = 2.11, compared with <40 years) was associated with a higher ICU mortality rate in 5183 patients treated with MV, which is in line with our finding.

In addition, the influence of sex on mortality rates in patients treated with MV is inconsistent. One study reported that women had a greater risk for in-hospital mortality [35], but another reported no such association [30]. In contrast, two large studies showed that being male predicted higher in-hospital mortality in patients treated with MV [36, 37]. Our study showed no significant differences in survival between the sexes, but the difference was significant after bias had been adjusted for. Differences in inclusion criteria, race, population-based cohorts, and geographic distribution make it difficult to generalize the effect of sex on critically ill patients.

Our study is also consistent with the literature, which shows that patients admitted to medical rather than surgical departments have more organ failures, and that comorbidities (e.g., DM, liver cirrhosis, cancer, stroke, and CHF) predicted worse outcomes. Manzano et al. found that a medical department admission was a significant predictor of mortality in patients requiring MV [38]. Other studies report that multiple organ failure is independently associated with mortality in patients with MV [30, 34, 39]. Similarly, the comorbidities of DM, CHF, stroke, liver cirrhosis, and cancer have been reported as independent predictors of short-term and long-term mortality in patients with MV [30, 32, 34, 38, 40, 41], which is consistent with our findings.

We also found that ESRD^Pos^ patients with MV were older, more often admitted to medical departments; and more often had comorbid DM, HTN, CAD, liver cirrhosis, and CHF than did ESRD^Neg^ patients before matching, although these factors could also be mortality contributors after matching. This was consistent with other studies [9, 10, 26]. In general, patients on long-term dialysis admitted to the ICU tend to have higher illness severity scores and multiple comorbidities, and to need more medical resources than does the general population [1].

### Strengths and limitations

Our study has some strengths. First, it is a large population-based analysis of the effect of ESRD on patients given a 1-MV, which includes patients not admitted to the ICU; this differentiates the present study from others. Second, the nationwide study design largely reduced the effect of referral bias, which is often seen in critical care studies.

Our study also has some limitations. First, all diagnoses, including comorbidities, relied on the claims data and ICD-9-CM diagnosis codes, which might lead to disease misclassification. Second, the NHIRD does not differentiate the stages of CKD in ESRD^Neg^ patients. Third, we were unable to take into account the illness severity scores of ESRD^Pos^ patients with MV because the data were unavailable; thus, we included the number of organ failures as a proxy for severity. Fourth, as in all observational studies, our study might contain some residual confounding; thus, it cannot show causality but only association between risk factors and mortality. Fifth, we excluded 146 ESRD^Pos^ patients (>10 %) because we were unable to match them with ESRD^Neg^ patients; this might contribute bias to our conclusions. Finally, not all patients were admitted to the ICU, which might have affected our evaluations of outcomes.

## Conclusions

Regardless of whether a patient is admitted to the ICU, being ESRD^Pos^ significantly increases the risk of death within 4 years after a 1-MV. After 4 years, however, survival rates are not significantly different. In addition to ESRD, older age, being male, being admitted by a medical department, multiple organ failure, and a history of comorbidities (DM, liver cirrhosis, cancer, stroke, and CHF) are associated with a higher mortality rate after a 1-MV. Physicians should keep these high-risk groups in mind and explain the prognosis to patients’ families when treating critical patients undergoing a 1-MV.

## Key messages

- ESRD-positive patients who underwent their first-ever mechanical ventilation were older, more often female, admitted to a medical department, had comorbid diabetes mellitus, hypertension, coronary artery disease, liver cirrhosis, and congestive heart failure than did ESRD-negative control patients before propensity score matching.
- After matching, the mortality rate of ESRD-positive patients was nearly twice as high as that of ESRD-negative patients.
- ESRD-positive patients had a significantly higher risk of death within 4 years after a first-ever mechanical ventilation, whether or not they were admitted to the intensive care unit.
- After 4 years of follow-up, however, there was no significant difference in mortality rates between ESRD-positive and ESRD-negative patients.
- In addition to ESRD, older age, being male, being admitted by a medical department, multiple organ failure, and a history of comorbid diabetes mellitus, liver cirrhosis, cancer, stroke, and congestive heart failure are associated with a higher mortality rate after a first-ever mechanical ventilation.

## Additional files

Additional file 1:
**ICD-9CM codes for comorbidities.** (DOCX 13 kb) Additional file 2:
**The 30-day, 6-month, and 1-, 2-, 5-, and 10-year survival rate differences in the ESRD** ^**Pos**^ **and ESRD** ^**Neg**^ **groups from the beginning.** (DOCX 17 kb) Additional file 3:
**Crude and adjusted hazard ratios (HR) of death in ESRD** ^**Pos**^ **patients treated with ventilation (derived from Cox proportional hazard regression models).** (DOCX 16 kb)

## Abbreviations

1-MV: first-ever mechanical ventilation
ARF: acute respiratory failure
CAD: coronary artery disease
CHF: congestive heart failure
CI: confidence interval
CKD: chronic kidney disease
COPD: chronic obstructive pulmonary disease
DM: diabetes mellitus
ESRD: end-stage renal disease
ESRD^Neg^: controls without ESRD
ESRD^Pos^: patients with ESRD
HR: hazard ratio
HTN: hypertension
ICD-9-CM: International Classification of Diseases, Ninth Revision, Clinical Modification
ICU: intensive care unit
IR: incidence rate
IRB: Institutional Review Board
IRR: incidence rate ratio
LHID2000: Longitudinal Health Insurance Database 2000
MV: mechanical ventilation
NHIRD: National Health Insurance Research Database
NHRI: National Health Research Institute
