# Supplementary material for: Effect of end-stage renal disease on long-term survival after a first-ever mechanical ventilation: a population-based study
Source: Crit Care. 2015 Oct 1;19:354. doi: 10.1186/s13054-015-1071-x (PMC4589902; doi:10.1186/s13054-015-1071-x)
Supplement: Additional file 1: — ICD-9CM codes for comorbidities. (DOCX 13 kb) [file 13054_2015_1071_MOESM1_ESM.docx]

| Additional file 1: ICD-9CM codes for comorbidities | |
| --- | --- |
| Diabetes | 250 |
| Hypertension | 401-405 |
| Coronary artery disease | 410-414 |
| Cirrhosis | 571.2, 571.5, 571.6, 789.5 , 456.20 |
| Chronic obstructive pulmonary disease | 490-496 |
| Cancer | 140-208 |
| Stroke | 430-436 |
| Congestive heart failure | 428 |
