# Supplementary material for: Effect of end-stage renal disease on long-term survival after a first-ever mechanical ventilation: a population-based study
Source: Crit Care. 2015 Oct 1;19:354. doi: 10.1186/s13054-015-1071-x (PMC4589902; doi:10.1186/s13054-015-1071-x)
Supplement: Additional file 2: — The 30-day, 6-month, and 1-, 2-, 5-, and 10-year survival rate differences in the ESRD Pos and ESRD Neg groups from the beginning. (DOCX 17 kb) [file 13054_2015_1071_MOESM2_ESM.docx]

**Additional file 2. The 30-day, 6-month, and 1-, 2-, 5-, and 10-year survival rate differences in the ESRD^Pos^ and ESRD^Neg^ groups from the beginning.**

| **Survival rate (%)** | | | | | | | |
| --- | --- | --- | --- | --- | --- | --- | --- |
| **Follow up endpoint** | **At risk (n)** | **30 days** | **6 months** | **1 year** | **2 years** | **5 years** | **10 years** |
| ESRD^Pos^ | 1185 | 58.14% | 40.31% | 36.52% | 32.56% | 26.62% | 22.41% |
| ESRD^Neg^ (Controls) | 9480 | 66.66% | 54.33% | 51.49% | 48.03% | 42.70% | 37.12% |

ESRD: end stage renal disease; ESRD^Pos^: with ESRD; ESRD^Neg^: without ESRD
