# Supplementary material for: Effect of end-stage renal disease on long-term survival after a first-ever mechanical ventilation: a population-based study
Source: Crit Care. 2015 Oct 1;19:354. doi: 10.1186/s13054-015-1071-x (PMC4589902; doi:10.1186/s13054-015-1071-x)
Supplement: Additional file 3: — Crude and adjusted hazard ratios (HR) of death in ESRD Pos patients treated with ventilation (derived from Cox proportional hazard regression models). (DOCX 16 kb) [file 13054_2015_1071_MOESM3_ESM.docx]

**Additional file 3. Crude and adjusted hazard ratios (HR) of death in ESRD^Pos^ patients treated with ventilation (derived from Cox proportional hazard regression models).**

| **Cohort** | **Crude HR (95% CI)** | **Adjusted HR (95% CI)** |
| --- | --- | --- |
| Age (years) |  |  |
| 0-50 | 1.00 | 1.00 |
| 50~64 | 1.32 (1.03-1.69) | 1.24 (0.97-1.59) |
| ≧65 | 1.80 (1.44-2.26) | 1.62 (1.28-2.05) |
| Sex |  |  |
| Female | 0.89 (0.78-1.01) | 0.87 (0.76-1.00) |
| Male | 1.00 | 1.00 |
| Comorbidity |  |  |
| Diabetes | 1.16 (1.01-1.32) | 1.13 (0.98-1.30) |
| Hypertension | 0.93 (0.81-1.06) | 0.93 (0.81-1.08) |
| Coronary artery disease | 1.09 (0.95-1.26) | 1.05 (0.91-1.22) |
| Liver cirrhosis | 1.29 (1.04-1.59) | 1.25 (1.00-1.55) |
| COPD | 1.35 (1.13-1.62) | 1.27 (1.06-1.53) |
| Cancer | 0.91 (0.76-1.10) | 1.03 (0.85-1.25) |
| Stroke | 1.27 (1.10-1.47) | 1.21 (1.04-1.41) |
| CHF | 0.98 (0.82-1.16) | 0.88 (0.74-1.06) |
| Department to which admitted |  |  |
| Surgery | 0.78 (0.67-0.91) | 0.90 (0.77-1.06) |
| Medical | 1.00 | 1.00 |
| Number of organ failures (other than lungs and kidneys) |  |  |
| 0 | 1.00 | 1.00 |
| 1 | 1.57 (1.35-1.82) | 1.39 (1.19-1.63) |
| ≧2 | 1.82 (1.14-2.91) | 1.89 (1.17-3.05) |
| Ventilator duration (days) (continuous) | 1.00 (1.00-1.00) | 1.00 (1.00-1.00) |
| ICU stay (days) (continuous) | 1.00 (0.99-1.00) | 1.01 (1.01-1.02) |
| Hospital stay (days) (continuous) | 0.99 (0.99-1.00) | 0.99 (0.98-1.00) |

HR: hazard ratio; ESRD: end-stage renal disease; ESRD^Pos^: with ESRD; COPD: chronic obstructive airway disease; CHF: congestive heart disease; ICU: intensive care unit.
